# Silver-Surface-Enhanced Raman Spectra of Berberine: Analyte-Induced Surface Changes, Variable Concentration Correlation, and Excitation Wavelength Dependence

**DOI:** 10.1021/acs.langmuir.4c05174

**Published:** 2025-06-24

**Authors:** Ivan Kopal, Valerie Smeliková

**Affiliations:** Department of Physical Chemistry, 52735 University of Chemistry and Technology Prague, Technická 5, 160 00 Prague 6, Czech Republic

## Abstract

The utilization of surface-enhanced Raman scattering (SERS) for the analysis of biologically important compounds is strictly dependent on the properties of the substance being analyzed. One of them is berberine, a highly valued bioactive alkaloid sourced from various botanical species, which is renowned for its multifaceted health-enhancing attributes, although its potential negative effects have been widely discussed. Here, we aimed to investigate the properties of berberine influencing the SERS intensity. By modifying silver colloids by the wide range of berberine concentrations, we have revealed its ability to significantly affect the nanoparticle surface’s properties, which results in complex concentration-dependent behavior. Characterization using extinction spectroscopy and transmission electron microscopy was performed to describe the ongoing effects. These results show that the tendency of silver nanoparticles to preferentially form assemblies with different geometries is the main reason for the nonlinear concentration dependence of the SERS signal. Additionally, we have investigated the effect of the excitation wavelength (532, 785, and 1064 nm) used. Such experiments not only provided the first comparison of the berberine SERS spectra measured with three different excitation wavelengths but also demonstrated that the observed intensity dependence is valid over a wide interval of excitation wavelengths. Apart from the physicochemical point of view, we also paid attention to effects important for possible analytical applications, such as reproducibility and long-term validity of the observed trends.

## Graphical abstract

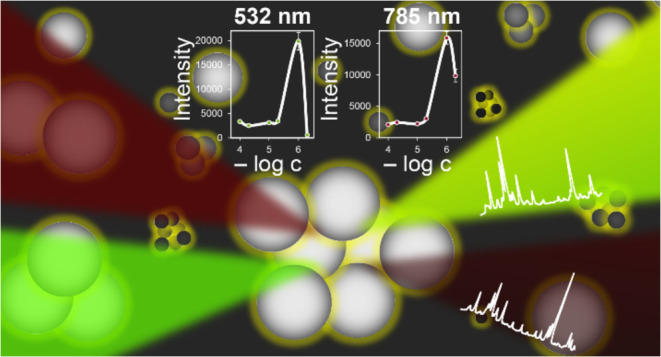

## Introduction

Berberine (BrBr), a natural alkaloid found in various plant species including those from the Berberidaceae, Fumariaceae, and Papaveraceae families, has captivated the scientific community and healthcare practitioners due to its diverse and promising array of potential health benefits. From its well-documented anti-inflammatory and antibacterial properties to its intriguing antitumor, cardiovascular, hypoglycaemic, lipid-lowering, antioxidant, and antiosteoporosis activities, berberine stands out as a versatile and multifunctional bioactive compound.^1–3^ However, to fully harness its potential and understand its intricate molecular behavior, the need for advanced analytical techniques has become increasingly evident. Previous studies have reported the analysis of berberine, using primarily LC,^4–7^ electrochemical methods,^8,9^ or spectrophotometric techniques.^10^ Surface-enhanced Raman scattering (SERS) spectroscopy, however, presents a compelling alternative due to its remarkable sensitivity and ability to detect biologically important compounds in low-concentration solutions.^11–18^

SERS has already been established as a cutting-edge analytical technique that combines the principles of Raman spectroscopy with the remarkable enhancement of signal intensity achievable through the specific properties of nanostructured surfaces.^19^ Although the amplified Raman scattering effect, arising from molecules in close vicinity of plasmonic metals, was first reported almost 50 years ago,^20^ it took the concerted effort of countless research groups to fully comprehend the various types of enhancing mechanisms at play.^21–24^ Usually, the largest part of the enhancement is caused by the so-called electromagnetic mechanism, which is dependent on localized surface plasmon resonance and is linked to the morphology and material properties of enhancing substrates as well as the excitation wavelength.^19,23,24^ One particularly important aspect influencing SERS efficiency is the presence of gap-plasmons in nanoassemblies, whose properties can be precisely tuned by controlling nanoparticle size, shape, and interparticle distance, either through simple principles or lithographic fabrication. By optimizing these parameters, the electromagnetic field enhancement can be maximized, leading to significantly improved Raman signal intensity.^25–29^ Another way to impact nanoparticle properties is the partial aggregation of a colloidal-enhancing substrate.^30,31^ This effect, described in the literature since the very beginning of SERS research, usually causes a red shift of the plasmon resonance maxima, thus aligning it with the excitation wavelength used.^32^ The aggregation itself may be induced by several factors, such as changes in pH,^33^ the addition of various salts,^34^ or the analyte itself.^35^

Usually, much less attention is paid to the chemical enhancement mechanism, which is related to, for example, the formation of surface complexes (molecule-metal). The properties of such modified nanostructured systems may significantly differ from those of the original substrates, thus leading to the observation of additional resonance effects. These situations may lead not only to further enhancement of the signal but may also greatly affect the optical response and the spectral profile of the investigated molecules.^19,23,24^

A comprehensive understanding of these physicochemical aspects of the SERS phenomenon was crucial in bringing this initially predominantly physical technique closer to modern analytical practice.^36–40^ Nowadays, the application of SERS in various fields of analytical chemistry is no longer surprising. This can be primarily attributed to the continuous advancement of novel amplifying substrates, which, alongside achieving the lowest possible detection limits, are also designed with an emphasis on universality and recyclability.^41–43^ Equally important in this context are studies focused on the application of SERS spectroscopy for the detection of biologically and environmentally relevant substances, often in complex matrices.^43–45^ To achieve optimal analytical conditions for such compounds, it is often beneficial to explore a wide range of excitation wavelengths, thereby maximizing the contribution of the various enhancement mechanisms involved.^46,47^ Therefore, a detailed investigation should precede all analytical applications, as the often-underestimated enhancement mechanisms may play a significant role depending on the nature of the studied compound and its interaction with the enhancing substrate.^48^ Furthermore, because of the gradual development of instruments, SERS is becoming a method suitable even for portable experimental setups, thus allowing fast and simple measurements.^49,50^

Here, we present the first complex study of BrBr’s concentration-dependent behavior when deposited on silver nanoparticles (AgNPs). Although some effort has already been made in this direction, most of the previously published results focused only on detection possibilities and vibrational assignments.^11,13,14^ The few studies that aimed for quantitative aspects were conducted in very narrow concentration ranges.^12,15,16^ To ensure that our results will be confidentially transferable to practical applications, we employed two portable Raman spectrometers with excitation wavelengths of 532 and 785 nm. To investigate signal behavior across a wider interval, we also performed our experiments on an FT-Raman spectrometer with an excitation wavelength of 1064 nm. Therefore, this study is also the first to compare the SERS spectra of berberine acquired by using three different excitation wavelengths. The use of multiple excitation wavelengths is crucial not only for assessing the contribution of individual enhancement mechanisms, whose differing effects typically manifest as changes in relative band intensities or even the presence or absence of specific bands when different lasers are employed.^46,47^ Moreover, employing various excitation wavelengths can also provide insight into the likelihood of fluorescence occurrence under a given light source, which represents a key factor for potential analytical applications. As the enhancing substrate, we used AgNPs reduced by hydroxylamine, as introduced by Leopold and Lendl. This procedure was selected due to its short synthesis time, robustness, and reliable enhancement properties.^51^ In addition to investigating concentration dependency, attention was paid to the formation of molecular complexes and colloidal aggregates, which had a significant impact on the resulting SERS intensity. We believe that these significant changes in AgNPs systems, which were characterized using electron microscopy and UV/vis spectroscopy, can not only improve the limit of BrBr’s detection but also make the described methodology more selective, providing invaluable when measuring more complex samples containing BrBr. Finally, we compared our Ag-SERS spectra with previously published results, thereby placing our findings in the broader context of the investigated problematics.^11–18^

## Experimental Section

### Materials

Berberine chloride (≥98.00%), silver nitrate (≥99.00%), and hydroxylamine hydrochloride (99.00%) were purchased from Sigma-Aldrich (Czech Republic). Sodium hydroxide (≥97.00%) was obtained from Penta (Czech Republic).

### Colloids Preparation

Silver colloidal nanoparticle solutions (AgNPs) were synthesized using the method described by Leopold N. and Lendl B. To prepare these solutions, 22.5 mL of a 3.33 × 10^–3^ mol/L NaOH solution was combined with an equal volume of 1.67 × 10^–3^ mol/L hydroxylamine hydrochloride solution. Subsequently, 5 mL of a 10^–2^ mol/L AgNO_3_ solution was rapidly introduced into the mixture under vigorous agitation. After a 10 min reaction period, the resulting product was a yellowish solution of silver nanoparticles with a pH of 7.^51^ These nanoparticles exhibited a spherical morphology with a mean diameter of 23 nm.^51^ For the preparation of BrBr-modified systems, all samples were prepared by adding 0.25 mL of BrBr solution at varying concentrations (2 × 10^–3^, 2 × 10^–4^, 2 × 10^–5^, 2 × 10^–6^ mol/L to obtain final sample concentrations of 10^–4^, 10^–5^, 10^–6^, 10^–7^ mol/L). These solutions were added to 4.75 mL of the prepared AgNPs. All subsequent measurements were conducted immediately after the addition of BrBr solution.

### Raman and SERS Spectroscopy

SERS spectra were acquired using dispersive instruments from B&W Tek: the iRaman Plus, featuring a diode laser emitting at a wavelength of 785 nm with a maximum power output of 350 mW (per sample after passing through fiber optics), and the iRaman Plus, equipped with a diode laser operating at 532 nm with a maximum power output of 30 mW (per sample after passing through fiber optics). The measurement range spans from 65 to 3200 cm^–1^ for the 785 nm laser and from 150 to 4200 cm^–1^ for the 532 nm laser. Both instruments offer a spectral resolution of 4.5 cm^–1^. The colloidal nanoparticle solutions were analyzed within a specialized sample compartment designed for liquid samples. Data acquisition was performed using BWSpec Software (B&W Tek). The laser power was set to 10% (approximately 35 mW) for the 785 nm laser and 100% (30 mW) for the 532 nm laser, with 10 acquisitions per measurement and an exposure time of 10 s for all measurements. The presented spectra were averaged and baseline-corrected using Omnic Software (Thermo Fisher Scientific).

Additional SERS spectra were obtained by a MultiRAM FT-Raman spectrometer (Bruker, USA and Germany). The instrument features a solid-state Nd:YAG laser (1064 nm) as the excitation source and is equipped with a highly sensitive Ge detector cooled with liquid nitrogen. The spectrometer’s measurement range extends from 50 to 3600 cm^–1^.

Measurements were performed using OPUS software with further data processing carried out in the previously mentioned OMNIC program. The colloidal solution was measured using a laser power of 300 mW, a resolution of 4 cm^–1^, and 512 scans.

### Extinction UV–vis Spectroscopy

The characterization of the prepared nanoparticles (NPs) was conducted using UV–vis spectroscopy, employing the CARY 50 single-beam spectrometer from Varian, USA. The measurements were carried out in the 300–900 nm range. A xenon discharge lamp operating in pulse mode served as the radiation source, and the spectrometer’s maximum scanning speed was 360 nm/min. Solution sampling was accomplished by using a 5 mm cuvette. Control and spectral recording were facilitated by Cary WinUV software.

### Transmission Microscopy

The aggregation of colloidal samples was investigated using an EFTEM 2200 FS (Jeol, Japan) transmission electron microscope (TEM). The 6 μL samples were deposited onto a 300-mesh Cu grid and measured immediately after drying. The image analysis was performed by using ImageJ software.

### Dynamic Light Scattering

Dynamic light scattering (DLS) measurements were performed using a Zetasizer Nano ZS ZEN3600 instrument (Malvern Panalytical, Great Britain) equipped with a 633 nm He–Ne laser as the light source. The scattered light was detected at an angle of 173° (backscatter configuration). Nanoparticle size estimation was based on optical parameters provided by the manufacturer (refractive index: *n* = 0.135; absorption, *k* = 3.99). Water was used as the dispersant (viscosity: η = 0.8872 cP; refractive index: *n* = 1.330). All measurements were conducted at 25 °C. For each sample, three series of 50 runs were performed, with each run lasting 10 s. The samples were measured in disposable polystyrene cuvettes (DTS0012), and all were diluted 2:5 with Milli-Q water to a final volume of 1.2 mL.

### Density Functional Theory Calculation

To assess the size of individual BrBr molecules, the molecular structure was optimized using Gaussian 16W software. The optimization was performed with the B3LYP functional and the 6-311G+(d,p) basis set. The dimensions were measured at the longest and widest parts of the molecule. The optimization was carried out in a default aqueous environment.

## Results and Discussion

In the following section of the text, we present SERS spectra obtained from molecules adsorbed onto the surfaces of AgNPs. The figures depict the averaged spectra derived from ten separate measurements performed on a single substrate. Notably, these spectra are showcased in offset mode, a deliberate choice aimed at accentuating variations in spectral intensity. In contrast, the extinction spectra are exhibited using a common-scale format for comparative purposes. The vibrational assignment of the Ag-SERS spectra was performed based on previously published data by Leona and Lombardi.^11^

As we already mentioned in the introduction, only a few works dealing with the SERS of BrBr have been published until now. Detailed information about individual experiments, namely, analyte concentration, excitation wavelength, and the enhancing substrate used, is listed in Table 1.

**Table 1:** Comparison of Previous Works Dealing with the SERS of BrBr

literature	enhancing substrate	excitation wavelength (nm)	concentration of BrBr (mol/L)	linear concentration range (mol/L)
Cañamares et al.^14^	citrate-reduced AgNPs^52^	1064	10^–5^	
Leona et al.^11^	citrate-reduced AgNPs^52^	785/1064	2.1 × 10^–5^/10^–5^	
Liu et al.^12^	citrate-reduced AgNPs^53^	633	1.5 × 10^–6^–3 × 10^–9^	1.5 × 10^–6^–3 × 10^–7^
				3 × 10^–7^–3 × 10^–9^
Zhao et al.^15^	HA reduced AgNPs^51^	633	9 × 10^–7^–5 × 10^–8^	5 × 10^–7^–5 × 10^–8^
Strekal et al.^13^	citrate-reduced AgNPs^52^/silver electrode	514	6 × 10^–6^/10^–5^	
Zhang et al.^16^	citrate-reduced AgNPs^52^	514	10^–5^–2 × 10^–6^ ^a^	

a BrBr was studied as a complex human serum albumin in different reactant ratios.

Most of the studies employed citrate-reduced^52^ nanoparticles except for Zhao et al.^15^ who used hydroxylamine-reduced nanoparticles as we did in our paper. The main difference between the studies mentioned is the excitation wavelength used. While the first two studies listed in the table worked with excitation wavelengths of 1064 and 785 nm, respectively, the next two worked with 633 nm, and the last-mentioned spectra were measured with a 514 nm laser. Leona et al.,^11^ Strekal et al.,^13^ and Cañamares et al.^14^ investigated BrBr at a single concentration. In contrast, Zhang et al.^16^ studied the concentration dependence but focused specifically on the concentration ratios of the BrBr–HSA complex (berberine with human serum albumin). A linear concentration dependence and the limit of detection (LOD) were reported by Liu et al.,^12^ who described an LOD of 3 × 10^–9^ mol/L and linearity in the ranges of 1.5 × 10^–6^–3 × 10^–7^ mol/L and 3 × 10^–7^–3 × 10^–9^ mol/L, respectively. Another report of linear dependence comes from Zhao et al.^15^ They state that a good linear relationship between intensity and concentration is observed when the concentration is below 5 × 10^–7^–5 × 10^–8^ mol/L. While it is apparent that most studies have focused on single-concentration measurements of BrBr, systematic investigations of its concentration dependence remain scarce. Unlike previous research, our study specifically addresses this gap by examining the concentration-dependent (10^–4^–10^–7^ mol/L) behavior of BrBr in detail.

In Figure 1a, Ag-SERS spectra of hydroxylamine-reduced silver nanoparticles modified by different concentrations of BrBr are shown. The spectra shown were measured by using an excitation wavelength of 785 nm. While the concentration dependence of the overall spectral intensity is observable to the naked eye, the spectral profile seems to be almost independent of BrBr’s concentration. One of the most intense spectral bands in the upper studied region is at 1566 cm^–1^ (in-plane ring deformation & OCH_3_ bending), followed by bands at 1443, 1422, 1398, and 1274 cm^–1^ (all of them related to in-plane deformations). The lower region is dominated by the “trident” of vibrations, with maxima located at 769 cm^–1^ (in-plane deformation and dioxolane OCH_2_O asymmetric stretching), 752 cm^–1^ (dioxolane and the nearest ring vibration, breathing), and 727 cm^–1^ (out-of-plane ring bending deformation).^11^

**Figure 1:**
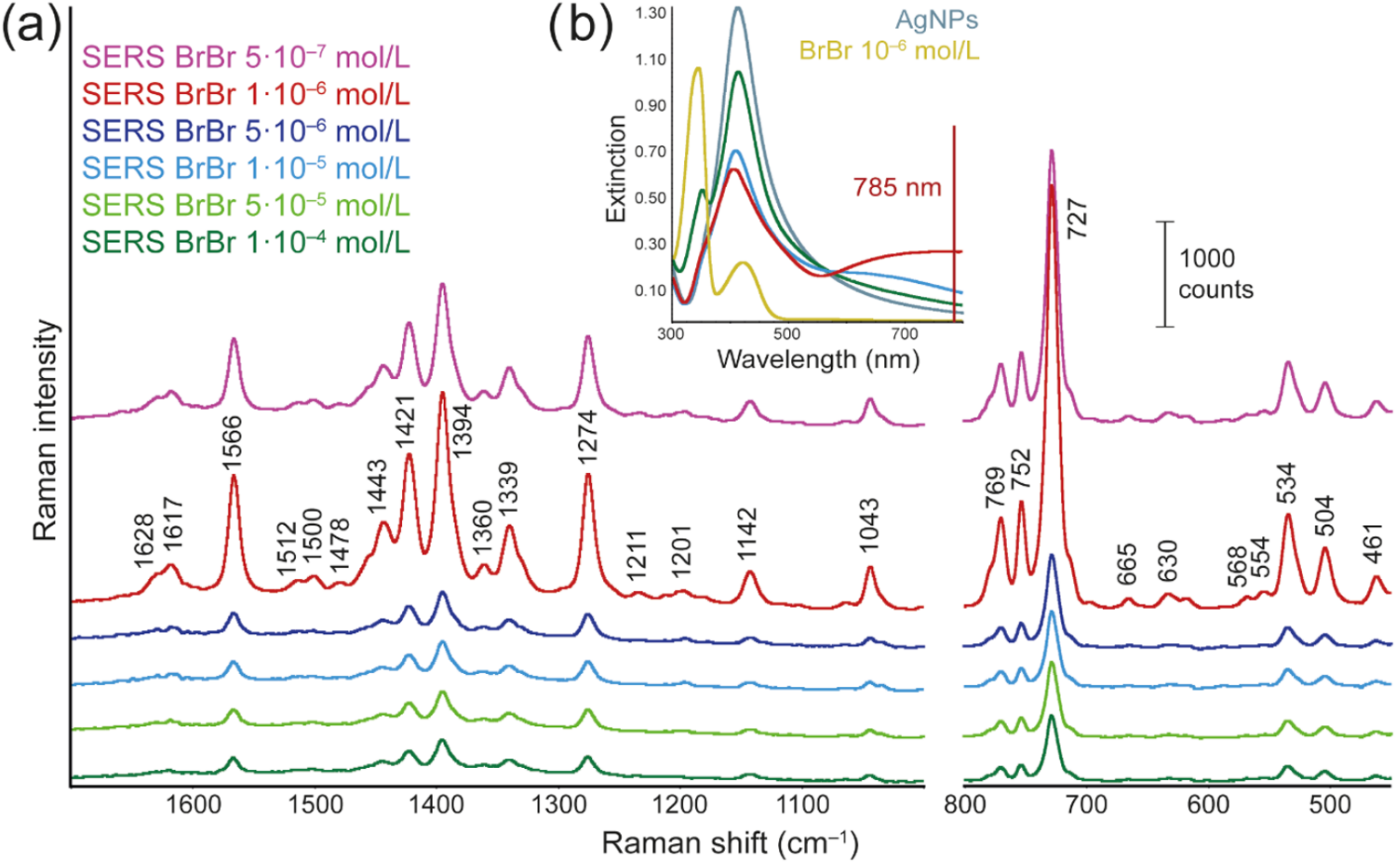
(a) Berberine Ag-SERS spectra measured with different BrBr concentrations. The shown spectra are averages of 10 independent spectra for each concentration. Spectra were measured using excitation wavelength of 785 nm and are displayed on an offset scale. (b) Extinction spectra of BrBr-modified AgNPs, pure AgNPs, and a pure BrBr solution. The scale is common for all spectra except for the BrBr’s solution, whose spectrum has been magnified.

It should be noted that the obtained spectra’s profiles are similar to the spectra previously published by Cañamares et al.,^14^ Leona et al.,^11^ Liu et al.,^12^ or Zhao et al.,^15^ but they differ significantly from the spectra published by Strekal et al.^13^ and Zhang et al.^16^ These two works were measured using an excitation wavelength of 514 nm. As for the first mentioned work, the difference is probably caused by the different experimental setup. Nevertheless, it is possible that different molecular orientation or another resonance effect is also the reason for such differences because the authors used citrate-reduced AgNPs, which may exhibit different resonance profiles. Also, the almost 20 nm difference between our lowest excitation wavelength (will be shown further in the text) and laser, which were used by Strekal et al.,^13^ may be sufficient to induce different resonances. As for the work of Zhang et al.,^16^ which focused on the possibility of berberine complexes with human serum albumin detection, the reason for observing a slightly different spectral profile is most likely due to the formation of the mentioned complexes.

For our systems, it seems that the overall spectral intensity remains almost the same across the BrBr concentration range 1 × 10^–4^–5 × 10^–6^ mol/L, followed by a steep increase in intensity for the BrBr concentration of 10^–6^ mol/L. The last measured SERS-active system, modified by the BrBr concentration of 5 × 10^–7^ mol/L, has a lower intensity than the previous one. This is in stark contrast to most traditional analytical techniques, where the analyte signal is simply proportional to the analyte’s concentration, and also to similar SERS experiments where the AgNPs used in this work were employed.^54^ Because of this, we first measured extinction spectra of the prepared modified substrates, which may carry valuable information about changes in the colloidal system or the occurrence of newly induced resonance effects. These spectra are displayed in Figure 1b. Here, the effect of BrBr concentration on the excitation spectra’s profile changes is undoubtedly observable. First, the position of the surface plasmon resonance (SPR) maximum changes. While the pure AgNPs exhibit a value of 414 nm, this gradually decreases in the modified systems to 413, 409, and 404 nm for BrBr concentrations of 10^–4^, 10^–5^, and 10^–6^ mol/L, respectively, as shown in Figure S1 (left). The main factors affecting the position of SPR are the material of nanostructure, shape and size of the nanoparticles, and the dielectric function of the surrounding. While the material of the nanoparticles remains the same, the size of the nanoparticles may be affected because of the ongoing aggregation, although such an effect is typically manifested via the presence of another extinction band. Therefore, we assume that the changes of position are caused by the different amount of BrBr molecules in the close vicinity of nanoparticles, which results in the changes of surroundings’ dielectric function. More importantly, the origin of higher-placed extinction maxima can be observed for the two lower displayed concentrations, i.e., 10^–5^ and 10^–6^ mol/L. This band, with maximum located approximately at 700 nm, increases in intensity when BrBr concentration decreases (Figure S1 (right)). This is extremely important because at least part of this band is located near the excitation wavelength (785 nm). This proximity may lead to an enhanced SERS signal due to possible resonance with the frequencies of this band. It seems that, in contrast to our previous work, this additional enhancement is related to the whole spectral interval, and thus, is most likely caused by a different effect.^41^ While in our previous article, the enhancement of the narrow spectral interval was caused by the formation of surface metal-molecule complexes, in the case of berberine, the origin is likely attributed to varying degrees of nanoassembly formation, as confirmed from the TEM images of the modified colloids (Figures 2 and S2).

**Figure 2:**
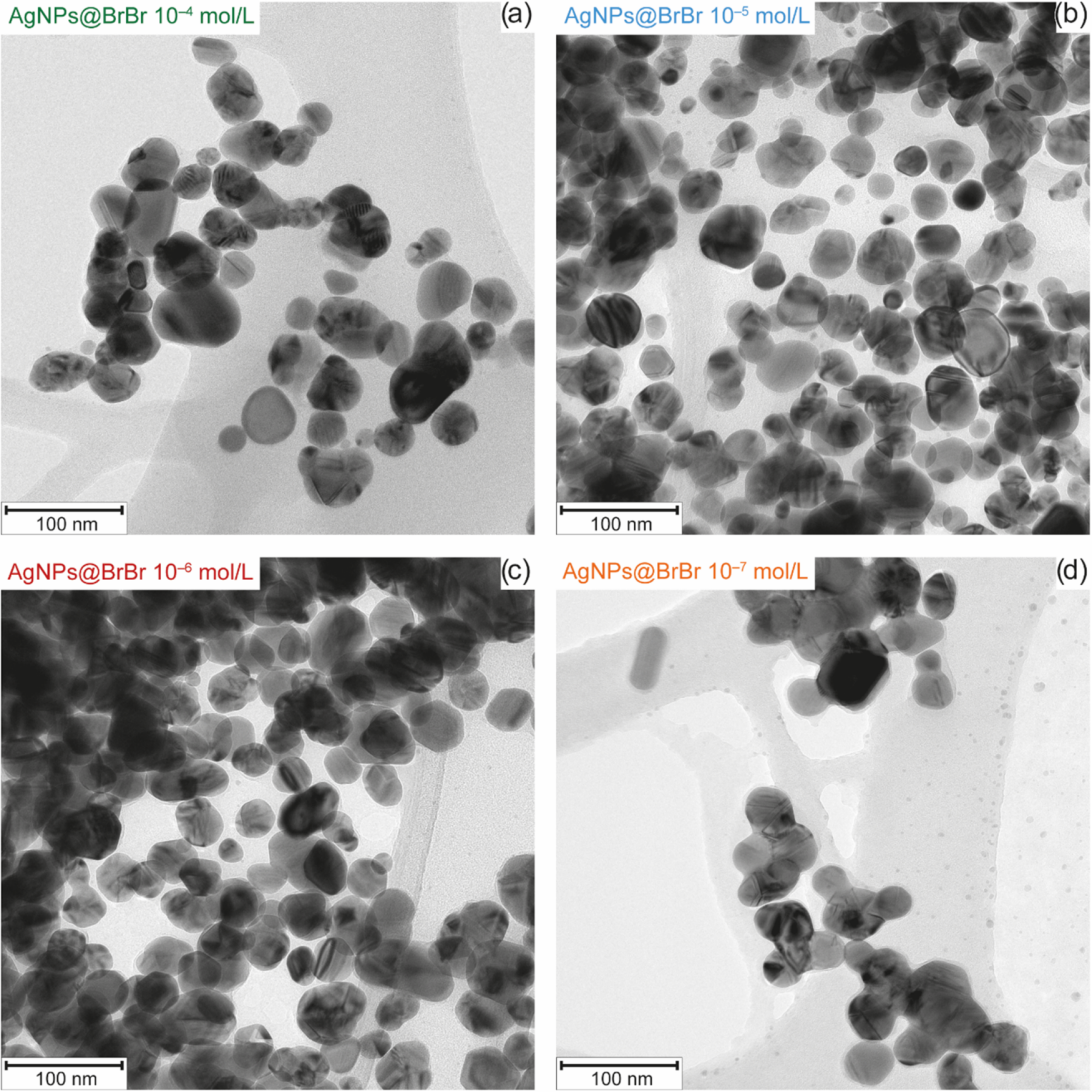
Transmission electron microscopy images of AgNPs modified by the BrBr concentration of (a) 10^–4^ mol/L, (b) 10^–5^ mol/L, (c) 10^–6^ mol/L, and (d) 10^–7^ mol/L.

Based on these images, it is suggested that while the BrBr concentration of 10^–4^ mol/L induces aggregation only slightly, modification by the following two lower concentrations (10^–5^ and 10^–6^ mol/L) leads to the highest level of aggregation. On the other hand, 10^–7^ mol/L concentrations do not appear to induce aggregation significantly, even when compared to the pure AgNPs sample (Figure S3). This suggests that the aggregation process is ongoing more effectively when some concentrations of BrBr are used, but this effect is nonlinear, which is also in agreement with the TEM image analysis performed (Figure S4). This could be possible because, for example, at a lower analyte’s concentration, there would be more nanoparticles available to aggregate, whereas when using a higher concentration, fewer nanoparticles capable of aggregation remain. In order to avoid potential distortion of the conclusions due to processes associated with sample drying, required for TEM but potentially leading to misleading interpretations of aggregation, we performed a similar experiment using dynamic light scattering. Particle size distributions obtained for systems modified by different concentrations of BrBr are displayed in Figure S5. These data indicate that, while systems with BrBr concentrations of 10^–4^ and 10^–7^ mol/L exhibit only fractions with diameters around 25 and 100 nm, the systems with 10^–5^ and 10^–6^ mol/L contain a significant proportion of particles with diameters between 200 and 500 nm. As no such fraction is present in the unmodified AgNP systems (Figure S6), it can be assumed that its presence in the aforementioned two systems results from aggregation, with the amount of aggregates being the highest in these two cases (Figure S7). This observation is in good agreement with the TEM results. As for the lowest concentration of BrBr (10^–7^ mol/L), whose particle distribution is very similar to unmodified AgNPs, no apparent SERS signal was observed (Figure S8a). This also correlates with the appearance of the extinction spectrum of this system, where no newly originated band is present (Figure S8b).

The different behavior of the AgNPs with respect to the concentration of berberine can also be explained by the varying state of AgNPs’ coverage. Using the simple idea that all nanoparticles are perfectly spherical with a diameter of 23 nm^51^ and that their concentration in the system is equal to 1.07 × 10^–10^ mol/L (based on the assumptions and calculations from our previous article^48^), the surface of one particle should be equal to 1661.903 nm^2^. Before the surface coverage of BrBr is estimated, it is necessary to investigate the molecular orientation with respect to the silver surface. The most enhanced vibrational band, located at 727 cm^–1^, is assigned to the out-of-plane ring bending deformation of the aromatic skeleton.^11^ Considering the surface selection rules, this vibrational mode likely occurs perpendicular to the surface, which explains its high intensity. Therefore, it can be inferred that BrBr molecules are oriented flat on the silver surface, which is also consistent with the observations made by Leona and Lombardi.^11^ Therefore, when approximating the BrBr molecule as a rectangle with dimensions of 1.5 × 0.6 nm (obtained from the optimized geometry using Gaussian, as described in the Experimental Section), the surface area occupied by a single molecule is estimated to be 0.9 nm^2^. This means that on average, there could be 1846 molecules present on one nanoparticle. Taking into account the computed concentration of the nanoparticles, full coverage would require the BrBr concentration of approximately 2 × 10^–7^ mol/L, meaning that our system with the lowest concentration would consist of nanoparticles not fully coated by BrBr. Using similar logic, systems with higher concentration would possess 5× (10^–6^ mol/L), 50× (10^–5^ mol/L), or 500× (10^–4^ mol/L) more BrBr molecules than what is needed for the full coverage of the nanoparticles surface. Additionally, the possibility that the surface charge is affected cannot be ruled out, as BrBr itself is a charged molecule. When modifying the systems with a concentration of 10^–7^ mol/L, the number of molecules would not be sufficient to cover nanoparticles entirely using the mentioned theory. The presence of NO_3_^–^ predominantly causes stability of the prepared AgNPs and OH^–^ anions on their surface.^51^ Although BrBr is a positively charged molecule, its low amount in this case may not be sufficient to compensate for the negative charge of nanoparticles and thus induce the formation of aggregates. This would correlate with both TEM and UV/vis of the mentioned system, where there were no signs of significant aggregation. Alternatively, using a BrBr concentration of 10^–6^ mol/L may be sufficient to compensate for the whole surface charge and thus cause significant aggregation process. The same would apply for the system modified by the concentration of 10^–5^ mol/L. In the UV/vis and TEM of these two systems, signs of the nanoassemblies’ formation can be found (extinction maxima around 700 nm and a high percentage of surface coverage, respectively). As for the highest examined concentration of BrBr, no distinct signs of aggregation were found at first sight. In the UV/vis, there is no apparent maximum around 700 nm, although the absolute intensity is increased to some extent when compared to that in the UV/vis of pure AgNPs. Also, the TEM images are quite similar to the systems modified by the concentration of 10^–7^ mol/L or to the pure nanoparticles. It can be speculated that the amount of positively charged BrBr molecules is so high that they effectively and rapidly cover the whole AgNPs area, and thus, they form the positively charged shell around nanoparticles, thus making them again stable to some extent. Although such reversion of NPs’ surface charge was reported before for some systems, it should be noted that the confirmation of this hypothesis would require additional experiments in the future.

The importance of the nanoassemblies’ formation, and especially of the sizes and shapes of the gaps formed, has been demonstrated previously, even in the case of different analytes.^26^ In the mentioned study, the authors employed so-called Soret colloids, which underwent a cooling procedure. As a result, the authors were able to create several fractions of differently assembled nanofeatures. Consequently, they optimized experimental factors to achieve the assembly of the most suitable features regarding the signal intensity and variability. Another study demonstrated that by varying the pH of the system, the minimalization of chemical enhancement can be achieved, which further improved the variability of the signal.^29^ Based on our observation, it is possible that BrBr concentration in the systems has a similar effect as the application of a thermal gradient, i.e., formation of defined nanoassemblies, in which different geometries result in the different enhancing properties. Unsurprisingly, with the proper geometry, not only Raman scattering, but also fluorescence signal can be enhanced, which may be undesirable if the detection of Raman scattering is aimed at.^27,28^ However, no such behavior was observed in our case.

As has already been stated, BrBr’s SERS signal in our systems is complexly proportional to the negative decimal logarithm of its concentration (−log *c*), as can be seen in Figure 3a, where the behavior of the band at 1274 cm ^–1^ is displayed. While for the systems modified by the BrBr concentration of 1 × 10^–4^–10^–5^ mol/L the signal level is almost the same, it is noticeably higher for the system modified by the concentration of 5 × 10^–6^ mol/L. This is followed by a significant increase in the concentration of 10^–6^ mol/L, while the last measured SERS-active system (with the BrBr concentration of 5 × 10^–7^ mol/L) has a lower intensity than the previous one. Such behavior was not recorded in the literature before, mostly because very few studies dealt with the concentration dependency of BrBr. The only two known to us, published by Liu et al.^12^ and Zhao et al.,^15^ investigated such behavior in narrower or different concentration intervals (1.5 × 10^–6^–3 × 10^–9^ mol/L and 9 × 10^–7^–5 × 10^–8^ mol/L, respectively). In the first case, the authors specifically claim that they observe two linear trends, ranging from 5 × 10^–6^ to 3 × 10^–7^ and from 10^–7^ to 3 × 10^–9^ mol/L. Focusing on the higher concentration interval, which can be directly compared to our results (although this study was measured using a 633 nm excitation wavelength), it can be seen that the points referring to the highest two concentration points are actually deviated from the linear trend noticeably. Based on our results, we believe that this is not a pure experimental error but that the system actually reaches a maximum of the dependence around the concentration of 10^–6^ mol/L, as can be seen in our data (the fact that in the case of mentioned study, this concentration is slightly different can be sufficiently explained by the usage of different AgNPs and different excitation laser also). As for the lower concentration range, we cannot directly compare our data because our detection limit is higher. Nevertheless, we have noticed that the spectral profile in Liu et al.’s^12^ work is different when reaching these lower concentrations. This could mean, for example, that the orientation of the molecules is different or that the authors detected indirect effect of the low berberine concentration on the other present species. As for the second mentioned study, which was actually performed with the same AgNPs (hydroxylamine-reduced) as we used, the observed trend is in agreement with ours in the lowest concentration region. Furthermore, authors specifically claim that when using higher concentration (around 10^–6^ mol/L), they have observed fast change in the colloids’ color. Therefore, they have ruled out this point from the calibration curve. We strongly believe that this system would be actually the most intense from the trend, as can be seen in our data. Also, their results confirm the idea that in some shorter concentration ranges, a linear dependence on the concentration can be achieved.

**Figure 3:**
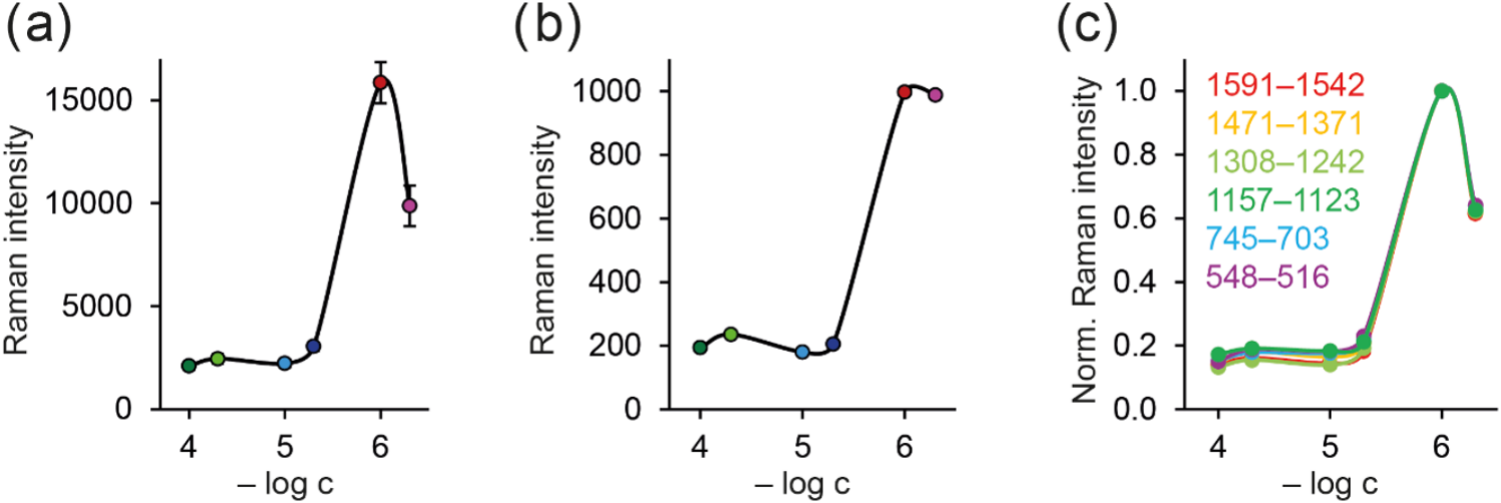
(a) Concentration dependence of SERS intensity in the interval 1308–1242 cm^–1^ taken as the area under the curve in a given spectral interval, (b) concentration dependency of the standard deviation, and (c) comparison of the concentration dependencies obtained from the six spectral regions and normalized to the maximum value of the trend. The values were obtained from the spectra measured with an excitation wavelength of 785 nm. Error bars represent the standard deviation from 10 measurements in both directions.

In Figure 3b, the dependence of the standard deviation on the concentration is displayed. It can be seen that it basically follows the intensity trend, i.e., that the higher the average intensity is, the higher the standard deviation of the concentration point. Nevertheless, the relative standard deviation in our experiments typically ranges between 5 and 10%, which is a good level for SERS detection. This implies all investigated bands across the wide spectral interval, as can be seen in Figure S9. To investigate the reproducibility of our observations, we also performed three independent sets of experiments. Our data (Figure S10) show that there is good agreement between the concentration dependencies recorded across different experiments. However, when aiming at the most accurate detection, it is undoubtedly desirable to perform analysis with the same AgNPs, which were used for the construction of the concentration dependency. Figure 3c shows normalized concentration dependencies for all investigated bands. It is apparent that the dependencies are very similar to each other. This means not only that basically any sufficiently intense and resolved band can be employed for analysis but also that the effect responsible for the nonlinear concentration dependency is affecting all bands equally. Therefore, this practically rules out the possibility of changing the BrBr orientation to the surface for different concentrations. If that were the case, due to the action of surface selection rules, the vibrations oriented perpendicularly to the surface would be the most enhanced one. Thus, if the BrBr orientation were different for some concentrations, the relative intensities would also be changed for those cases, and thus the shapes of concentration dependencies would also be affected.

Subsequently, we have calculated the analytical enhancement factor (AEF) values from eq 1:

1 $$\mathrm{AEF}=\frac{I_{\mathrm{SERS}}/c_{\mathrm{SERS}}}{I_{\mathrm{RS}}/c_{\mathrm{RS}}}$$

where *I*_SERS_, *I*_RS_, *c*_SERS_, and *c*_RS_ refer to the intensity (*I*) or concentration (*c*) of the SERS or Raman spectrum (RS), respectively.^19^ The concentration of BrBr solution was 2 × 10^–3^ mol/L, and the spectrum of this solution was measured 3 times using the same measurement conditions as for the SERS measurements. This allowed us to compute the value of AEFs for 3 data sets. For the selected interval (1471–1371 cm^–1^, as it was the only observable band in the nonenhanced BrBr solution’s spectra), we computed the value of AEF using the area of the mentioned band for all ten nonaveraged spectra (of the selected concentration) and the areas of the corresponding band in the pure BrBr spectra. Then, we performed this procedure with the second spectrum of BrBr and so on. In the end, we obtained 30 values of AEF, whose average and standard deviation values are presented in Figure S11a. Generally, using the AEF, we obtained values as high as 2.5 × 10^5^ for the two lowest concentrations, but significantly lower values for the systems containing higher amounts of berberine, which agrees with the observed SERS spectra. The exponential dependency seems to be the best for a description of the AEF concentration dependency in this case. Relatively lower values of AEF, which are sometimes expected to be higher, are largely caused by the definition of AEF itself. Although this enhancement factor is widely used for the comparison of individual substrates, it simultaneously uses several simplifications that do not always fully correspond to reality. Probably the most serious of them is that it uses the whole analyte concentration in the sample for SERS for the AEF estimation. However, it is known that only a fraction of the molecules present in the solution are often adsorbed onto the enhancing substrate, and therefore, only these molecules are majorly contributing to the observed signal. Although also molecules which are not in direct contact with the surface may contribute to the overall intensity, as the electromagnetic enhancement does not directly require the sorption of the molecules, its effect diminishes with the third power of the distance between the molecule and surface. Therefore, the contribution of the nonsorbed molecules is often negligible. We have already estimated the approximate concentration of the BrBr, which should be sufficient for the complete coverage of all AgNPs’ surface as 2 × 10^–7^ mol/L. If we considered this value as the relevant concentration of the SERS-contributing molecules for all systems, we would achieve enhancement factor values (marked as an EF in this case) between 10^5^ and 10^6^ for the systems with all BrBr concentrations (Figure S11b), which should be closer to reality. Unsurprisingly, as the concentration value is fixed at the concentration mentioned before, the concentration trend follows the SERS intensity trend.

To investigate the time stability of the prepared systems, we performed a series of experiments after 24 h. These results can be seen in Figures 4a and S12. Although it is obvious that the SERS intensity of the systems decreased over time, it is also apparent that all systems are SERS-active after at least 24 h following the addition of BrBr to the systems. The exact amount of the signal change can be seen in Figure 4b, where the ratio between the 1274 cm ^–1^ band area obtained after 0 and 24 h for different concentrations is displayed. It can be stated that the age of modified AgNPs affects the highest and the lowest concentrations (10^–4^ and 5 × 10^–7^ mol/L), while the intensity of the system with the BrBr concentration of 10^–5^ mol/L seems to be almost unchanged over time. This would probably mean that the stability of the different systems is affected by the number of BrBr molecules present, which is in agreement with our data presented so far. Nevertheless, normalized concentration dependence is still exhibiting similar courses even after 24 h (Figure 4c), which would suggest that the presented systems can be employed for the quantitative analysis even 24 h after the preparation. However, it should be noted that the concentration dependence should be obtained from systems of the same age as the colloid used for the measurement of the analyzed sample to prevent inclusion of unwanted errors into the results.

**Figure 4:**
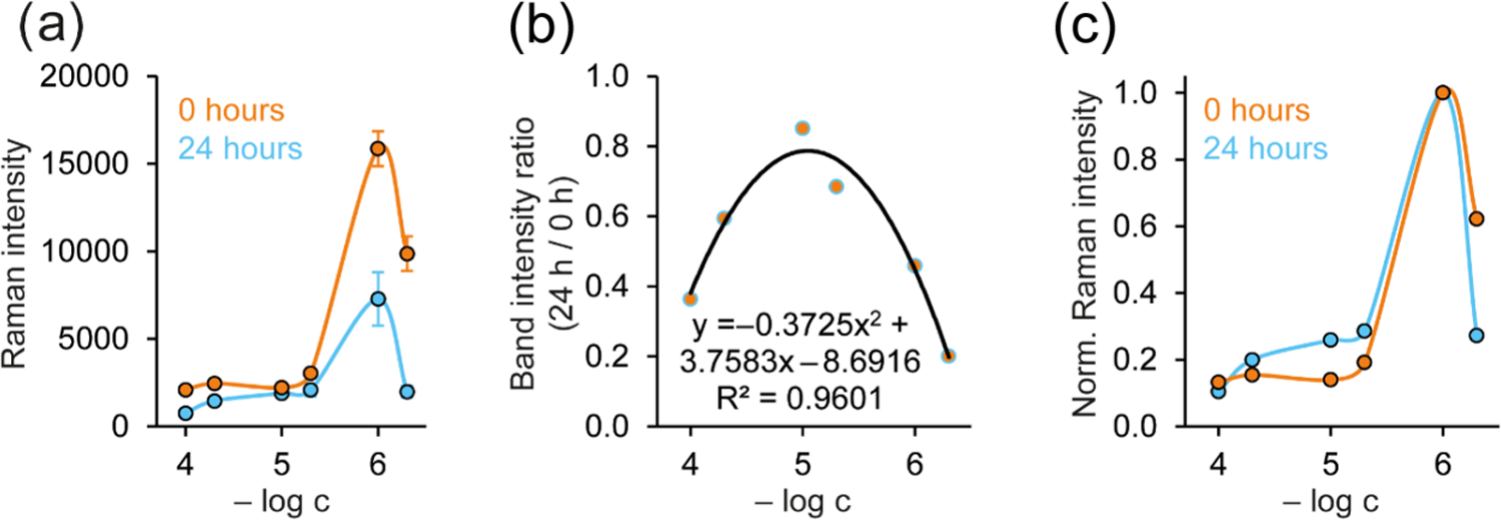
(a) Comparison of the concentration dependencies in the interval 1308–1242 cm^–1^ obtained during measurements after 0 and 24 h, (b) concentration dependency of the 1274 cm ^–1^ band intensity ratio (24/0 h), (c) comparison of the concentration dependencies in the interval 1308–1242 cm^–1^ obtained at the 0 and 24 h normalized to the maximum value of the trend. The values were obtained from the spectra measured with an excitation wavelength of 785 nm. Error bars represent the standard deviation from 10 measurements in both directions.

The dependence of the spectra appearance on the excitation wavelength is given in Figure 5a, while the spectra obtained for different concentrations using excitation wavelengths of 532 and 1064 nm can be seen in Figures S13 and S14, respectively. Although all obtained spectra are relatively similar, some differences may be observed. For example, the relative intensities of the bands around 730 cm^–1^ are notably different for the spectrum obtained using an excitation wavelength of 532 nm, where the 729 cm^–1^ band dominates the surrounding interval. Also, the relative intensities of the bands at 1044, 1276, 1340, and 1456 cm^–1^ (values refer to the bands in the 532 nm excited spectrum) are apparently changing in accordance with the laser selection. Furthermore, it can be generally stated that the relative intensity of all of the named bands increases proportionally to the value of the excitation wavelength. There could be several reasons for such observations, the direct determination of which is beyond the scope of this work. It can be suggested that the varying effect of the individual enhancing mechanisms is the main candidate, although it is difficult to predict which mechanism is causing such differences in this case, as the different degree of resonances may occur because of both the presence of aggregates (with the extinction maxima around 700 nm) or some chemical effect, such as charge transfer. However, the concentration dependencies obtained using different excitation wavelengths (Figure 5b–d) are generally very similar to each other. Therefore, it can be assumed that the presented trends are valid for all investigated excitation wavelengths, which may have implications for analytical chemistry. The analysis can be performed using various excitation wavelengths, which may be especially useful when there are undesired spectral interferences (such as mainly fluorescence but also possible resonance Raman scattering from the different species present in the samples) when using some of the excitation wavelengths. If that is the case, the choice of a different excitation wavelength may solve such an issue quickly while maintaining the specifics of concentration dependency.

**Figure 5:**
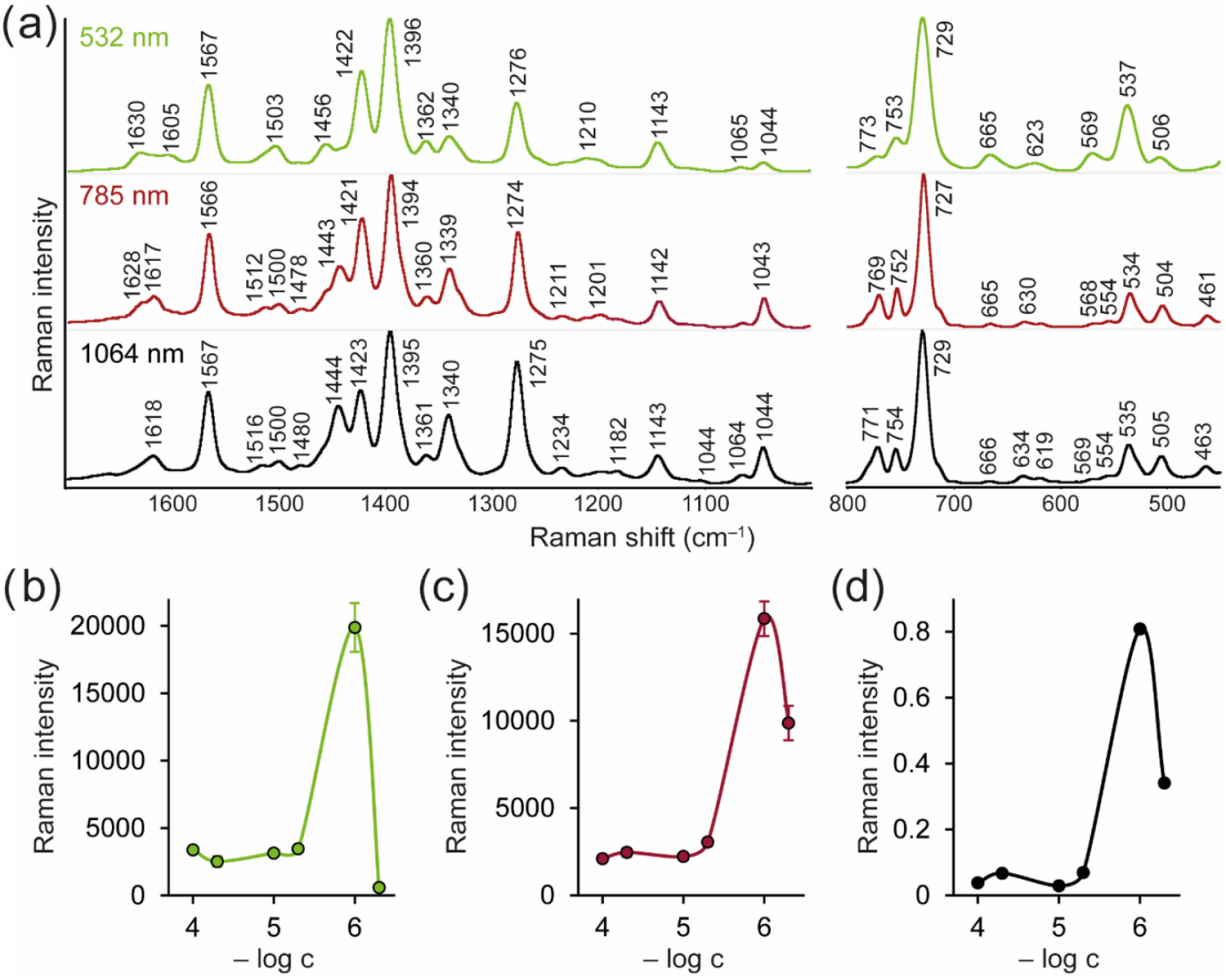
(a) Ag-SERS spectra of the system modified by the BrBr concentration of 10^–6^ mol/L measured with different excitation wavelengths and concentration dependence of SERS intensity in the interval 1308–1242 cm^–1^ taken as the area under the curve in a given spectral interval measured with the excitation wavelength of (b) 532 nm, (c) 785 nm, and (d) 1064 nm. Error bars represent the standard deviation from 10 measurements in both directions.

The concentration-dependent enhancement factors computed for excitation wavelengths of 532 nm (Figure S15) and 1064 nm (Figure S16) show a trend very similar to that observed with excitation at 785 nm. Specifically, systems with BrBr concentrations of 10^–6^ and 5 × 10^–7^ mol/L exhibit the highest enhancement values across all excitation wavelengths tested. However, while the overall trend is similar across excitation wavelengths, the absolute values of the enhancement factors differ depending on the excitation wavelength used. When enhancement factors are calculated based on the theoretical concentration required for full surface coverage—rather than the total BrBr concentration in the system—the highest enhancement factors are observed for excitation at 785 nm (Figure S17). This is likely caused by resonance with the newly emerged extinction band associated with the formation of larger aggregates, which has its maximum located between the excitation wavelengths of 532 and 1064 nm, making it most resonant with the 785 nm source. Therefore, this additional resonance is either absent or only weakly present at the other two excitation wavelengths. These findings imply that excitation at 785 nm is likely to provide the highest enhancement across the entire BrBr concentration range when such resonance conditions are met.

When comparing various analytical methods for berberine detection, one of the commonly used techniques is liquid chromatography (LC) coupled with a UV detector. However, this approach does not achieve sufficiently low limits of detection (LOD).^7^ To obtain high sensitivity, LC combined with mass spectrometry (LC-MS) is required. This technique can reach a limit of quantification (LOQ) as low as 3 × 10^–15^ mol/L.^6^ However, such low detection limits come at the cost of high instrument expenses, particularly due to the mass spectrometer detector. Gas chromatography (GC) is generally not used for berberine analysis due to its unsuitable physicochemical properties, which necessitate complex sample preparation, including derivatization. Another widely used method is high-performance liquid chromatography (HPLC), where LOD values are typically in the range of 10^–8^ mol/L,^4,7^ which is already comparable with the LOD achievable using SERS. Quantification of BrBr was also demonstrated using cyclic voltammetry, where the LOD reaches values around 10^–6^ mol/L.^8,9^ The same applies for fluorescence spectroscopy, in which it is also necessary to convert BrBr into the form of a suitable complex. Therefore, it can be said that while most analytical methods focus only on quantitative evaluation, SERS, on the other hand, offers the possibility to obtain additional information about the system, while maintaining the option of affordable and fast analysis when using portable Raman spectrometers.

Furthermore, better LODs can be achieved even for SERS after further future optimizations. Thus, further investigations should focus on exploring the aggregation mechanism (creation of nanoaggregate/nanoassembly/soret/cryosoret nanoassemblies driven by plasmonic hotspots) in greater detail to better understand its impact on SERS enhancement. Additionally, testing alternative enhancing substrates, such as gold nanoparticles or large-area SERS-active materials as well as modifying the reducing agent used in nanoparticle synthesis, could provide valuable insights into optimizing signal intensity and selectivity. Finally, extending this approach to structurally similar compounds to berberine could further validate the methodology and expand its applicability in analytical studies. Additionally, for such applications, it is essential to evaluate the influence of the matrix in the specific context and to consider the presence of potential interferents.

## Conclusions

In this study, we present SERS spectra of AgNPs systems modified by different concentrations of BrBr. The results show that the obtained concentration dependence of the SERS signal varies between inversely correlated, correlated, or almost independent in accordance with the actual concentration interval. while the largest SERS signal was observed at the lowest BrBr concentration of 10^–6^ mol/L. This effect correlates with the measured extinction spectra and TEM images of both modified and original AgNPs systems. These data confirmed the theory that BrBr concentration affects the properties of the prepared Ag sols, resulting in the formation of different aggregates/nanoassemblies, thereby enhancing both incidental and scattered radiation intensity through additional resonance effects in the modified systems. Based on these findings, we plotted the concentration dependency curves of different vibrational bands (or groups of bands). The resulting dependencies of SERS intensity on the logarithm of concentration were found to be the same, meaning that our observations are valid for the whole spectral interval, while the standard deviation of the observed spectra ranges between 5 and 10%.

Additionally, we have investigated the excitation wavelength dependency using three different lasers (532, 785, and 1064 nm). Based on these results, it can be stated that although there are subtle deviations in the obtained spectral profiles, the overall concentration dependency is the same for all of the named excitation wavelengths. This fact has consequences for analytical use because, if necessary, it allows for performing an analysis with a different laser while preserving the quantitative character of the dependence.

Our results provide an example of the colloidal systems being affected by the variable concentration of the model probe, resulting in the atypical behavior of the SERS signal. Aside from the physicochemical point of view, this observation supports the idea that the changes caused by the model probes’ concentration could be more suitable for the semiquantitative analysis than the SERS-signal concentration dependency itself. Furthermore, our results show that in the cases where the detection of berberine is aimed at the SERS can be successfully employed across several orders of magnitude in concentration.

## Supplementary Material



## Notes

UV–vis extinction spectra’s trends; TEM and DLS results and analysis; AgNPs@BrBr 10^–7^ mol/L spectra (Raman and UV/vis extinction); concentration dependences of SERS intensity and RSD in other spectral intervals; reproducibility information; enhancement factors; time stability; and 532 and 1064 nm excitation (PDF)

## References

[1] Preininger, V. Chemotaxonomy of Papaveraceae and Fumariaceae. In Alkaloids Chemistry and Biology; Wiley, 1986; Vol. 29, pp 1–98.

[2] Chahine J., Saffon N., Cantuel M., Fery-Forgues S. (2011). Spontaneous
Formation of Fluorescent Nanofibers and Reticulated Solid from Berberine
Palmitate: A New Example of Aggregation-Induced Emission Enhancement
in Organic Ion Pairs. Langmuir..

[3] Kong W., Wei J., Abidi P., Lin M., Inaba S., Li C., Wang Y., Wang Z., Si S., Pan H., Wang S., Wu J., Wang Y., Li Z., Liu J., Jiang D. J. (2004). Berberine is a novel cholesterol-lowering
drug working
through a unique mechanism distinct from statins. Nat. Med..

[4] Ge A.-H., Bai Y., Li J., Liu J., He J., Liu E.-W., Zhang P., Zhang B.-L., Gao X.-M., Chang Y.-X. (2014). An activity-integrated
strategy involving ultra-high-performance liquid chromatography/quadrupole-time-of-flight
mass spectrometry and fraction collector for rapid screening and characterization
of the α-glucosidase inhibitors in *Coptis chinensis* Franch. (Huanglian). J. Pharm. Biomed. Anal..

[5] Liu F., Li Z., Shi X., Zhong M. (2011). Determination of berberine, palmatine
and jatrorrhizine in rabbit plasma by liquid chromatography–electrospray
ionization-mass spectrometry. J. Pharm. Biomed.
Anal..

[6] Kim J. H., Mai X.-L., Kim K. Y., Sim M.-S., Lee S.-Y., Seo H.-W., Lee G., Kim D.-J., Kim K. H. (2019). A Sensitive
and Rapid LC-MS/MS Method for Determination of Berberine in Human
Plasma. Spectrosc. Lett..

[7] Wang J., Jiang Y., Wang B., Zhang N. (2019). A review on analytical
methods for natural berberine alkaloids. J.
Sep. Sci..

[8] Geto A., Pita M., De Lacey A. L., Tessema M., Admassie S. (2013). Electrochemical
determination of berberine at a multi-walled carbon nanotubes-modified
glassy carbon electrode. Sens. Actuators, B.

[9] Skopalová J., Vacek J., Papoušková B., Jirovský D., Maier V., Ranc V. (2012). Electrochemical oxidation
of berberine
and mass spectrometric identification of its oxidation products. Bioelectrochemistry.

[10] Fan S., Lu Z., Yan Z., Hu L. (2024). Interactions of three berberine mid-chain
fatty acid salts with bovine serum albumin (BSA): Spectroscopic analysis
and molecular docking. Int. J. Biol. Macromol..

[11] Leona M., Lombardi J. R. (2007). Identification of berberine in ancient and historical
textiles by surface-enhanced Raman scattering. J. Raman Spectrosc..

[12] Liu J., Liu W., Huang Y., Zhao X., Feng Z., Wang D., Gong Z., Fan M. (2021). Self-supporting liquid film as reproducible
SERS platform for therapeutic drug monitoring of berberine hydrochloride
in human urine. Microchem. J..

[13] Strekal N. D., Motevich I., Nowicky J., Maskevich S. (2007). IR absorption
and surface-enhanced Raman spectra of the isoquinoline alkaloid berberine. J. Appl. Spectrosc..

[14] Cañamares M. V., Lombardi J. R., Leona M. (2008). Surface-enhanced Raman
scattering
of protoberberine alkaloids. J. Raman Spectrosc..

[15] Zhao J., Liu Y., Fales A. M., Register J., Yuan H., Vo-Dinh T. (2014). Direct analysis
of traditional Chinese medicines using surface-enhanced Raman scattering
(SERS). Drug Test. Anal..

[16] Zhang W., Zhao Y., Bai X., Hui G., Lombardi J. R., Zhao D., Zhao B. (2011). The auxiliary determination
of the
binding site of berberine binding to human serum albumin by surface-enhanced
Raman scattering. Vib. Spectrosc..

[17] Lin Y.-K., Tai R.-J., Wei S.-C., Luo S.-C. (2020). Electrochemical
SERS on 2D Mapping for Metabolites Detection. Langmuir.

[18] Šloufová I., Šlouf M., Vlčková B., Gajdošová V., Zedník J., Vohlídal J. (2019). Controlled Tuning of the Size of
Ag-Hydrosol Nanoparticles by Nonstabilized THF and Detection of Peroxides
in THF. Langmuir.

[19] Procházka, M. Surface-Enhanced Raman Spectroscopy; Springer, 2016.

[20] Fleischmann M., Hendra P. J., McQuillan A. J. (1974). Raman spectra of pyridine adsorbed
at a silver electrode. Chem. Phys. Lett..

[21] Langer J., Jimenez de Aberasturi D., Aizpurua J., Alvarez-Puebla R. A., Auguié B., Baumberg J. J., Bazan G. C., Bell S. E., Boisen A., Brolo A. G. (2020). Present and future of
surface-enhanced Raman scattering. ACS Nano.

[22] Moskovits M., Piorek B. D. (2021). A brief history
of surface-enhanced Raman spectroscopy
and the localized surface plasmon Dedicated to the memory of Richard
Van Duyne (1945–2019*)*. J. Raman Spectrosc..

[23] Lombardi J. R., Birke R. L. (2009). A unified view of surface-enhanced Raman scattering. Acc. Chem. Res..

[24] Lombardi J. R., Birke R. L. (2008). A unified approach
to surface-enhanced Raman spectroscopy. J. Phys.
Chem. C.

[25] Hong J., Kawashima A., Hamada N. (2017). A simple fabrication of plasmonic
surface-enhanced Raman scattering (SERS) substrate for pesticide analysis
via the immobilization of gold nanoparticles on UF membrane. Appl. Surf. Sci..

[26] Moronshing M., Subramaniam C. (2018). Room Temperature, Multiphasic Detection
of Explosives,
and Volatile Organic Compounds Using Thermodiffusion Driven Soret
Colloids. ACS Sustainable Chem. Eng..

[27] Bhaskar S., Moronshing M., Srinivasan V., Badiya P. K., Subramaniam C., Ramamurthy S. S. (2020). Silver Soret Nanoparticles for Femtomolar Sensing of
Glutathione in a Surface Plasmon-Coupled Emission Platform. ACS Appl. Nano Mater..

[28] Cheerala V. S. K., Ganesh K. M., Bhaskar S., Ramamurthy S. S., Neelakantan S. C. (2023). Smartphone-Based Attomolar Cyanide
Ion Sensing Using
Au-Graphene Oxide Cryosoret Nanoassembly and Benzoxazolium-Based Fluorophore
in a Surface Plasmon-Coupled Enhanced Fluorescence Interface. Langmuir.

[29] Moronshing M., Bhaskar S., Mondal S., Ramamurthy S. S., Subramaniam C. (2019). Surface-enhanced Raman scattering
platform operating
over wide pH range with minimal chemical enhancement effects: Test
case of tyrosine. J. Raman Spectrosc..

[30] Meyer M., Le Ru E. C., Etchegoin P. G. (2006). Self-Limiting
Aggregation Leads to
Long-Lived Metastable Clusters in Colloidal Solutions. J. Phys. Chem. B.

[31] Bell S. E. J., McCourt M. R. (2009). SERS enhancement
by aggregated Au colloids: effect
of particle size. Phys. Chem. Chem. Phys...

[32] Fornasiero D., Grieser F. (1987). Analysis of the visible
absorption and SERS excitation
spectra of silver sols. J. Chem. Phys..

[33] Kerker M., Siiman O., Wang D. S. (1984). Effect
of aggregates on extinction
and surface-enhanced Raman scattering spectra of colloidal silver. J. Phys. Chem. A.

[34] Feilchenfeld H., Siiman O. (1986). Surface Raman excitation
and enhancement profiles for
chromate, molybdate, and tungstate on colloidal silver. J. Phys. Chem. A.

[35] Sánchez-González R., Silva V., Suazo C., Soto J. P., Sanchez-Cortes S., Campos-Vallette M., Leyton P., Imbarack E. (2022). SERS study on the aggregation
mechanisms resulting from the orientation of dipyridinic derivatives
on gold nanoparticles. Spectrochim Acta, Part
A.

[36] Dendisová M., Palounek D., Švecová M., Prokopec V. (2019). SERS study
of fluorescent and non-fluorescent flavonoids: what is the role of
excitation wavelength on SERS optical response?. Chem. Pap..

[37] Kopal I., Švecová M., Plicka M., Dendisová M. (2023). Time dependent
investigation of copper colloids SERS-activity. Mater. Today Commun..

[38] Fornasaro S., Alsamad F., Baia M., Batista
de Carvalho L. A., Beleites C., Byrne H. J., Chiadò A., Chis M., Chisanga M., Daniel A. (2020). Surface
enhanced Raman spectroscopy for quantitative analysis: results of
a large-scale European multi-instrument interlaboratory study. Anal. Chem..

[39] Tuckmantel
Bido A., Azarakhshi A., Brolo A. G. (2022). Exploring Intensity Distributions
and Sampling in SERS-Based Immunoassays. Anal.
Chem..

[40] Fan M., Andrade G. F., Brolo A. G. (2020). A review on recent advances in the
applications of surface-enhanced Raman scattering in analytical chemistry. Anal. Chim. Acta.

[41] Novoa-De
León I. C., Johny J., Vázquez-Rodríguez S., Avellaneda-Avellaneda D., Shaji S., Sepúlveda-Guzmán S. (2025). Nanocarbon
Hybrid Films of Reduced Graphene Oxide and N-Doped Graphene Quantum
Dots as a Metal-Free Platform for Graphene-Enhanced Raman Scattering. ACS Appl. Mater. Interfaces..

[42] Pavlović M., Khomiakova N., Kočišová E., Procházka M., Kylián O. (2025). Sers-active and recyclable Nb2O5/Au
platforms. Mater. Lett..

[43] Xin L., Liu Y., Wang L., Li Z. (2025). Graphene-based SERS-active substrates
for environmental detection: nanoarchitectonics and applications. J. Mater. Sci..

[44] Xia L., Yu H., Li Y. (2024). Comprehensive Application and Prospects
of Surface-Enhanced
Raman Spectroscopy in Natural Product Research. Crit. Rev. Anal. Chem..

[45] Zhang A., Ding Z., Shen Z., Yan Z., Han K., Li J., Zhang M., Zhang W. (2025). Nano-arrayed
Cu2S@MoS2 heterojunction
SERS sensor for highly sensitive and visual detection of polystyrene
in environmental matrices. Talanta.

[46] Kopal I., Švecová M., Král M., Michalcová A., Lapčák L., Matějka P., Dendisová M. (2025). Exploring the variability of methylene
blue′s
surface-enhanced Raman scattering spectra – Impact of the experimental
conditions (Plasmonic metal and excitation wavelength) and ongoing
reactions (Reduction and photon-induced demethylation). Colloids Surf., A.

[47] Klenotová M., Matějka P. (2025). SERS analysis
of saliva and its key components: The
effects of various collection methods, sample dilution, excitation
wavelengths, and enhancing substrates. Vib.
Spectrosc..

[48] Smeliková V., Kopal I., Člupek M., Dendisová M., Švecová M. (2024). Unveiling the Crucial Role of Chemical
Enhancement in the SERS Analysis of Amphetamine–Metal Interactions
on Gold and Silver Surfaces: Importance of Selective Amplification
of the Narrow Interval of Vibrational Modes. Anal. Chem..

[49] Zhang D., Tang L., Chen J., Tang Z., Liang P., Huang Y., Cao M., Zou M., Ni D., Chen J., Yu Z., Jin S. (2021). Controllable Self-Assembly
of SERS Hotspots in Liquid Environment. Langmuir.

[50] Huang Q., Gong H., Wang G., Hu W., Wang W., Pan S., Xu J., Liu G., Tian Z. (2024). Positively Charged
Silver and Gold Nanoparticles with Controllable Size Distribution
for SERS Detection of Negatively Charged Molecules. Langmuir.

[51] Leopold N., Lendl B. (2003). A new method for fast
preparation of highly surface-enhanced Raman
scattering (SERS) active silver colloids at room temperature by reduction
of silver nitrate with hydroxylamine hydrochloride. J. Phys. Chem. B.

[52] Lee P. C., Meisel D. (1982). Adsorption and surface-enhanced
Raman of dyes on silver
and gold sols. J. Phys. Chem. A.

[53] Sun J., Gong L., Lu Y., Wang D., Gong Z., Fan M. (2018). Dual functional PDMS
sponge SERS substrate for the on-site detection
of pesticides both on fruit surfaces and in juice. Analyst.

[54] EL-Zahry M. R., Refaat I. H., Mohamed H. A., Rosenberg E., Lendl B. (2015). Utility of surface enhanced Raman
spectroscopy (SERS) for elucidation
and simultaneous determination of some penicillins and penicilloic
acid using hydroxylamine silver nanoparticles. Talanta.

