# Supplementary material for: Silver-Surface-Enhanced Raman Spectra of Berberine: Analyte-Induced Surface Changes, Variable Concentration Correlation, and Excitation Wavelength Dependence
Source: Langmuir. 2025 Jun 24;41(26):16725–35. doi: 10.1021/acs.langmuir.4c05174 (PMC12257584; doi:10.1021/acs.langmuir.4c05174)
Supplement: Supplementary file 1 [file la4c05174_si_001.pdf]

Supporting Information

Silver-Surface-Enhanced Raman Spectra of Berberine: Analyte-Induced Surface Changes, Variable Concentration Correlation, and Excitation Wavelength Dependence

Ivan Kopal* ‡ and Valerie Smeliková ‡

Department of Physical Chemistry, University of Chemistry and Technology Prague, Technická 5, 160 00 Prague 6, Czech Republic

‡ Both authors contributed equally

* Corresponding author: tel.: + 420 220 444 040, e-mail: kopali@vscht.cz

Table of Contents

1. Surface plasmon extinction trends S2
2. Transmission electron microscopy images and analysis S2-S3
3. Dynamic light scattering analysis S4-S5
4. SERS of AgNPs modified by the BrBr concentration of 10^{-7} mol/L S5
5. Concentration dependencies in different spectral regions S6
6. Reproducibility of the concentration dependencies S6
7. Enhancement factors (excitation wavelength of 785 nm) S7
8. Time stability of the spectral response S8
9. SERS spectra recorded using excitation wavelengths of 532 nm and 1064 nm . S8-S9
10. Enhancement factors (excitation wavelengths of 532 nm and 1064 nm) S9-S10
11. Comparison of enhancement factors S10

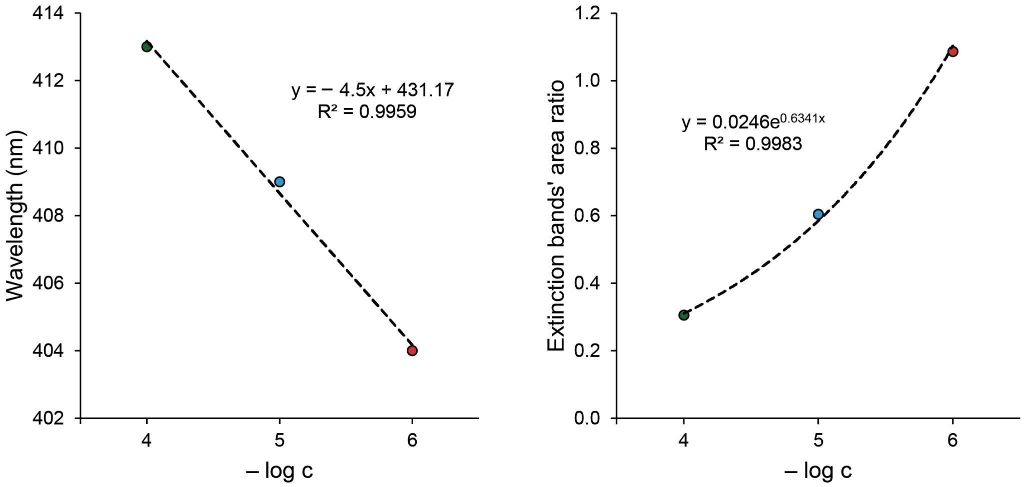

Figure S1. Concentration dependence of the main surface plasmon resonance maxima (**left**) and of the extinction bands' area ratio (**right**).

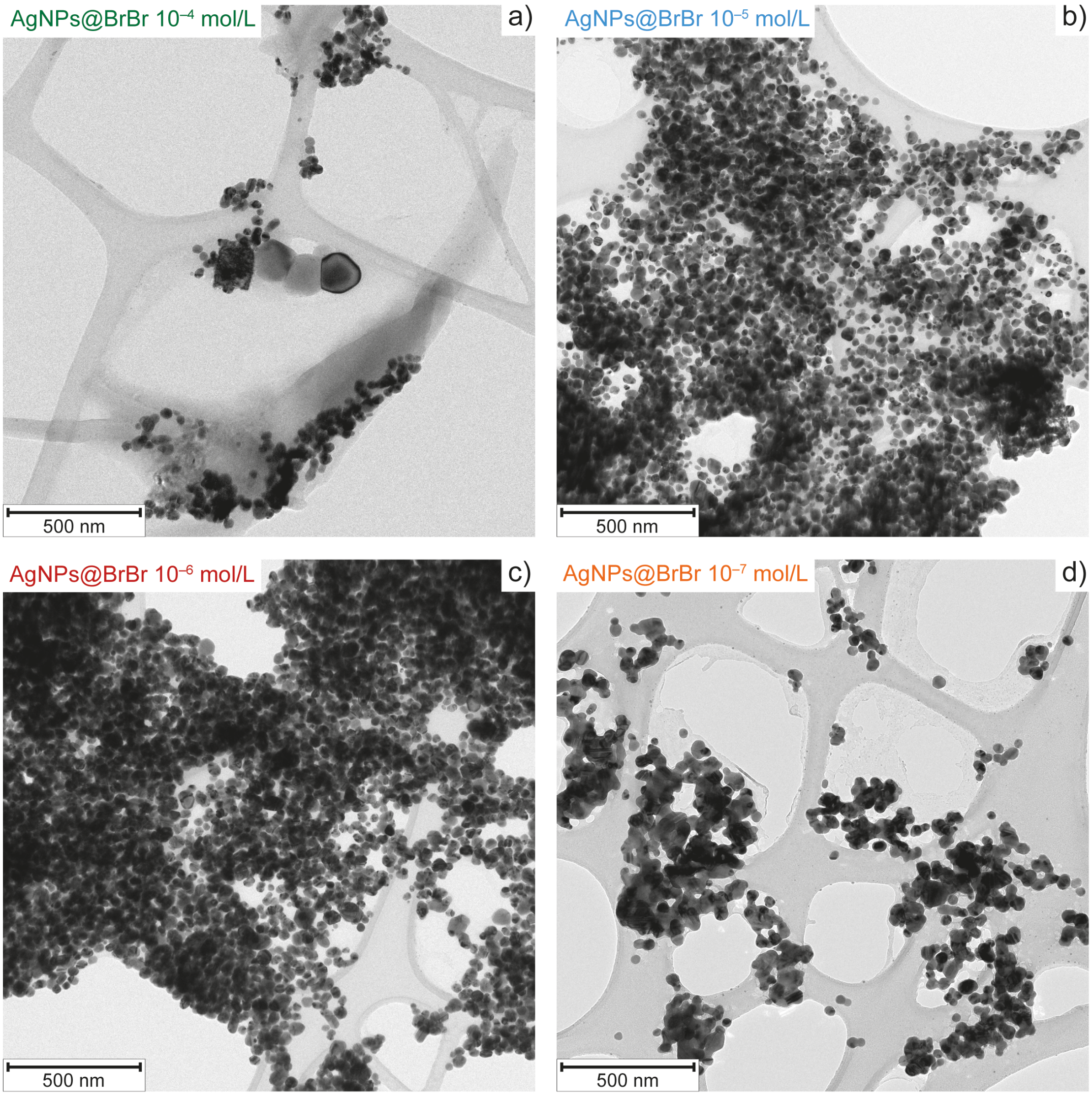

Figure S2. Transmission electron microscopy images of AgNPs modified by the BrBr concentration of **a)** 10^{-4} mol/L, **b)** 10^{-5} mol/L, **c)** 10^{-6} mol/L and **d)** 10^{-7} mol/L.

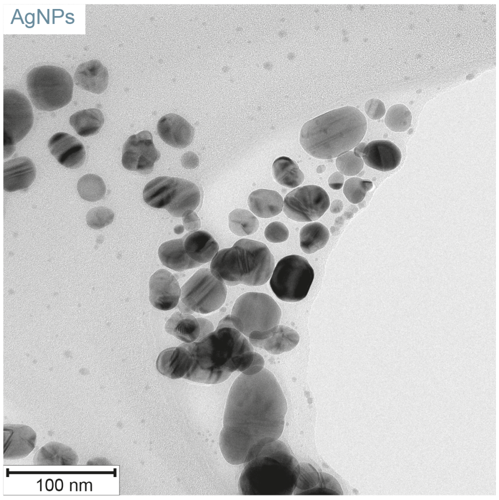

Figure S3. Transmission electron microscopy images of the pure AgNPs.

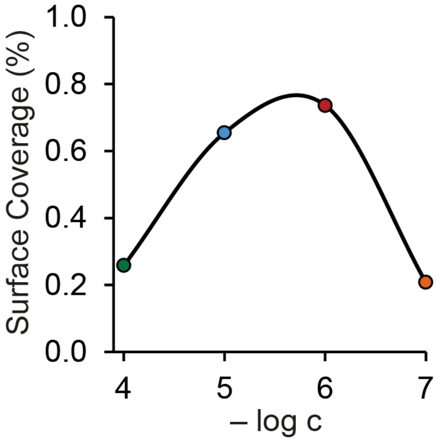

Figure S4. Concentration dependence of the surface coverage (%) obtained from the TEM image analysis according to the Eq. S1.

$$\text{Surface Coverage} = \left(\frac{A_{\text{NPs}}}{A_{\text{Image}}}\right) \cdot 100 \qquad \text{Eq. S1}$$

A_{NPs} – Image area covered by nanoparticles

A_{Image} – Image area

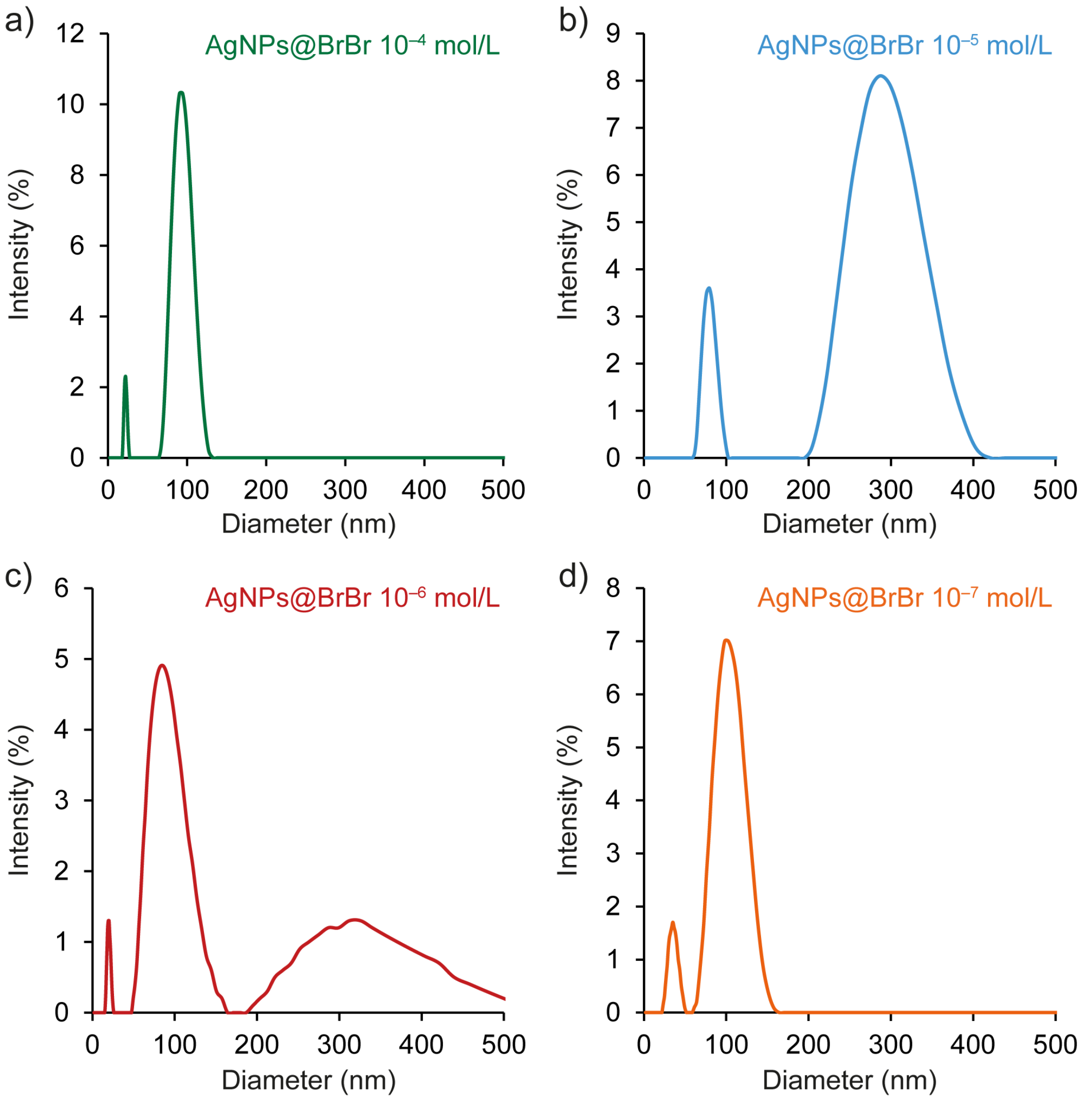

Figure S5. DLS-measured particle size distributions of the systems modified by the BrBr concentration of a) 10^{-4} mol/L, b) 10^{-5} mol/L, c) 10^{-6} mol/L and d) 10^{-7} mol/L.

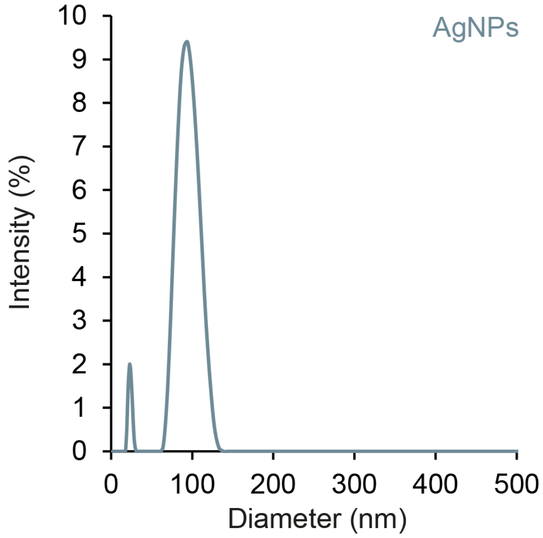

Figure S6. DLS-measured particle size distributions of the unmodified AgNPs.

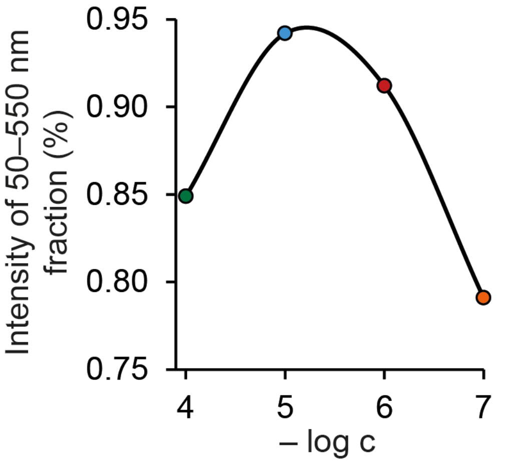

Figure S7. DLS-derived intensity of the 50–550 nm size fraction as a function of BrBr concentration.

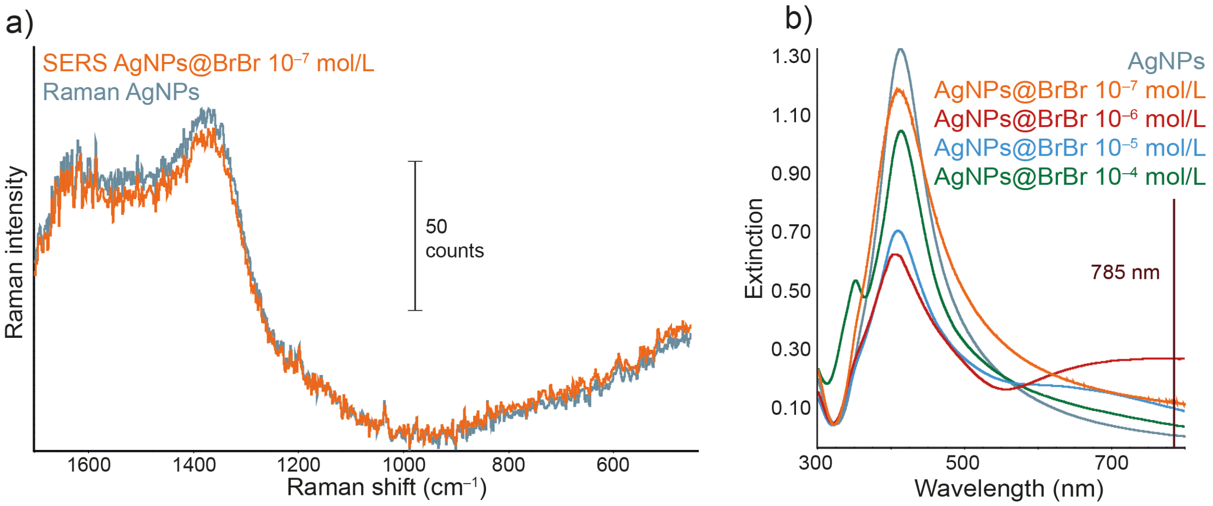

Figure S8. Comparison of the **a)** SERS spectra of BrBr (10^{-7} mol/L) and the Raman spectra of pure AgNPs **b)** Extinction UV/Vis spectra of pure and modified AgNPs.

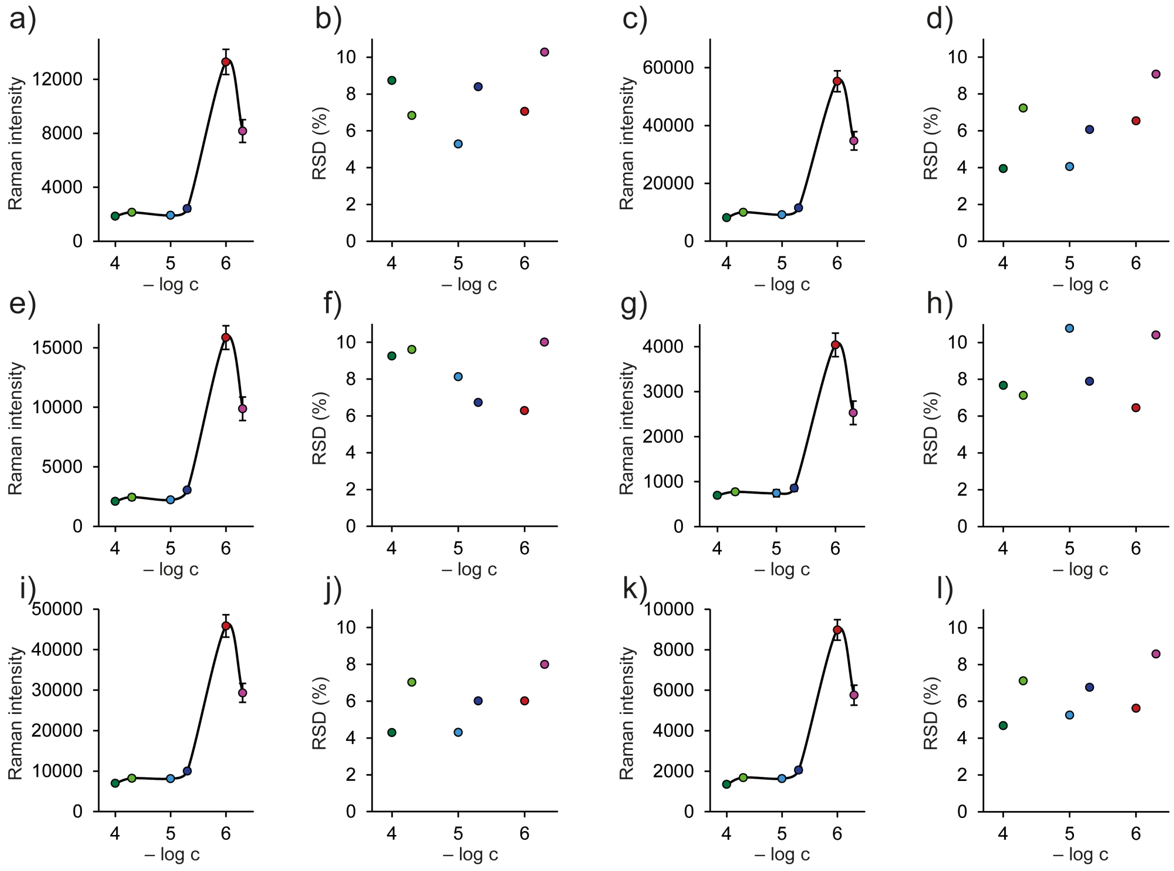

Figure S9. Concentration dependence and relative standard deviation (RSD) of SERS intensity (taken as the area under the curve in a given spectral interval) in the interval 1591–1542 cm^{-1} (**a**), **b**)), 1471–1371 cm^{-1} (**c**), **d**)), 1308–1242 cm^{-1} (**e**), **f**)), 1157–1123 cm^{-1} (**g**), **h**)), 745–703 cm^{-1} (**i**), **j**)), 548–516 cm^{-1} (**k**), **l**)). The values were obtained from the spectra measured with an excitation wavelength of 785 nm. Error bars represent the standard deviation from 10 measurements in both directions.

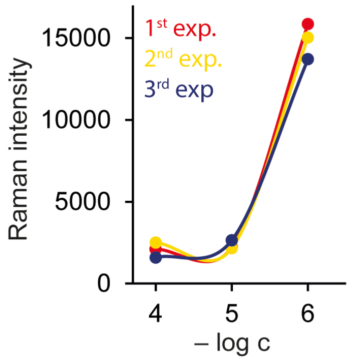

Figure S10. Concentration dependence of SERS intensity in the interval 1308–1242 cm^{-1} (taken as the area under the curve in a given spectral interval) obtained during three independent sets of experiments. The values were obtained from the spectra measured with an excitation wavelength of 785 nm.

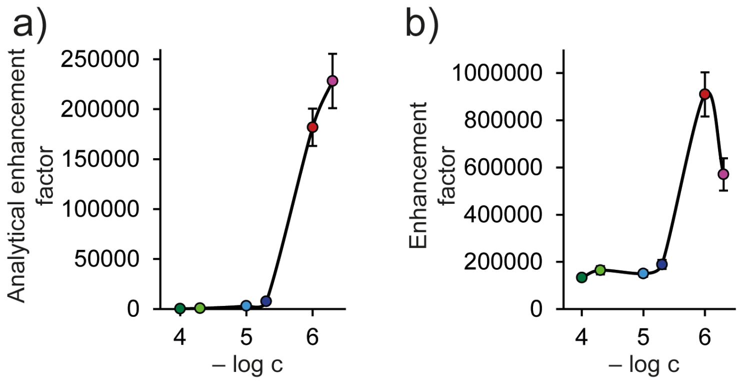

Figure S11. Concentration dependence of the enhancement factors values obtained from the Eq. 1 when using **a)** entire added concentrations of the BrBr, **b)** theoretical concentration value required for the complete surface coverage. The values were obtained from the spectra measured with an excitation wavelength of 785 nm. Error bars represent the standard deviation from all computations in both directions.

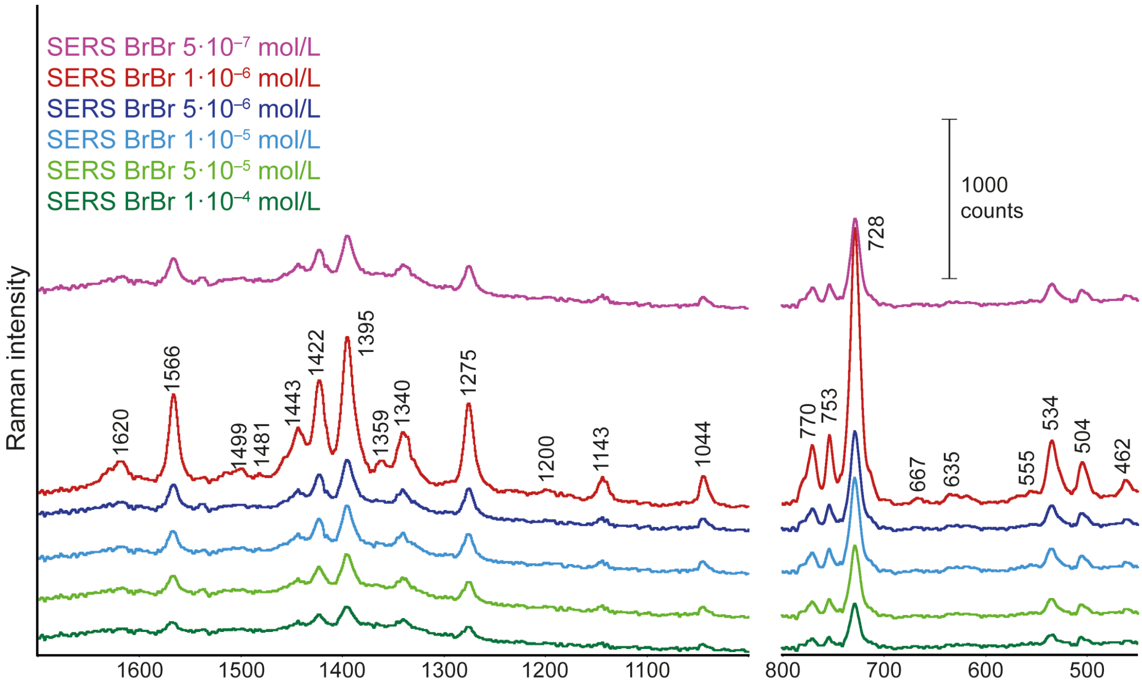

Figure S12. Berberine Ag-SERS spectra measured with different BrBr concentrations after 24 hours since the BrBr addition to the solutions. The shown spectra are averages of five independent spectra for each concentration. Spectra were measured using excitation wavelength of 785 nm and are displayed on an off-set scale.

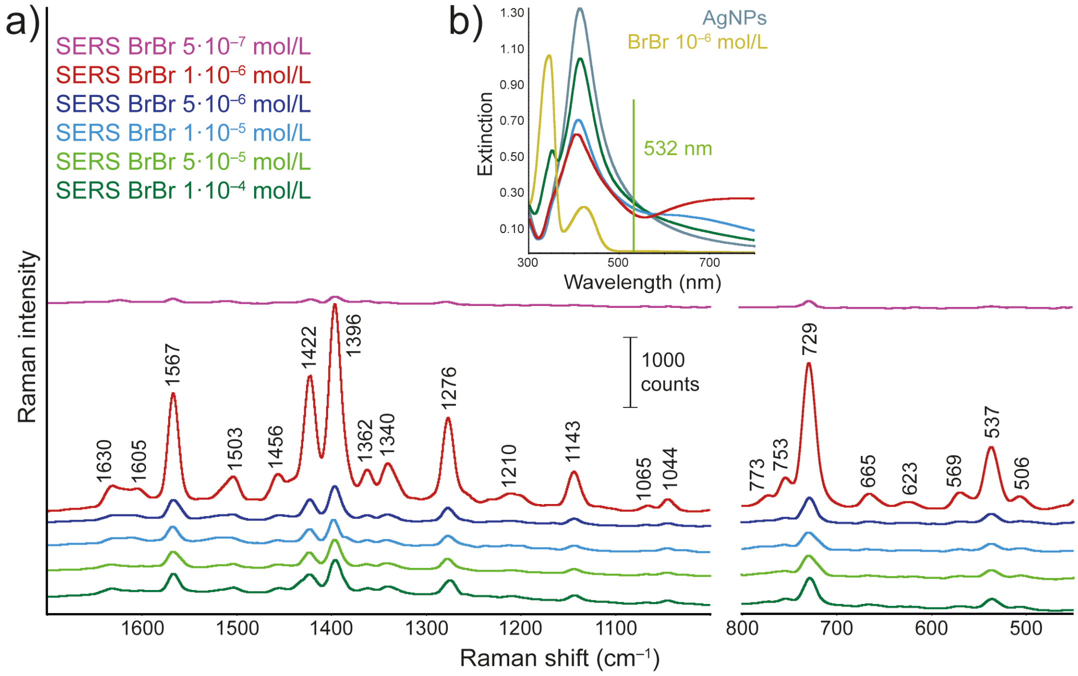

Figure S13. a) Berberine Ag SERS spectra measured with different BrBr concentrations. The shown spectra are averages of ten independent spectra for each concentration. Spectra were measured using excitation wavelength of 532 nm and are displayed on an off-set scale, **b)** Extinction spectra of BrBr-modified AgNPs, pure AgNPs, and a pure BrBr solution. The scale is common for all spectra except for the BrBr's solution, whose spectrum has been magnified.

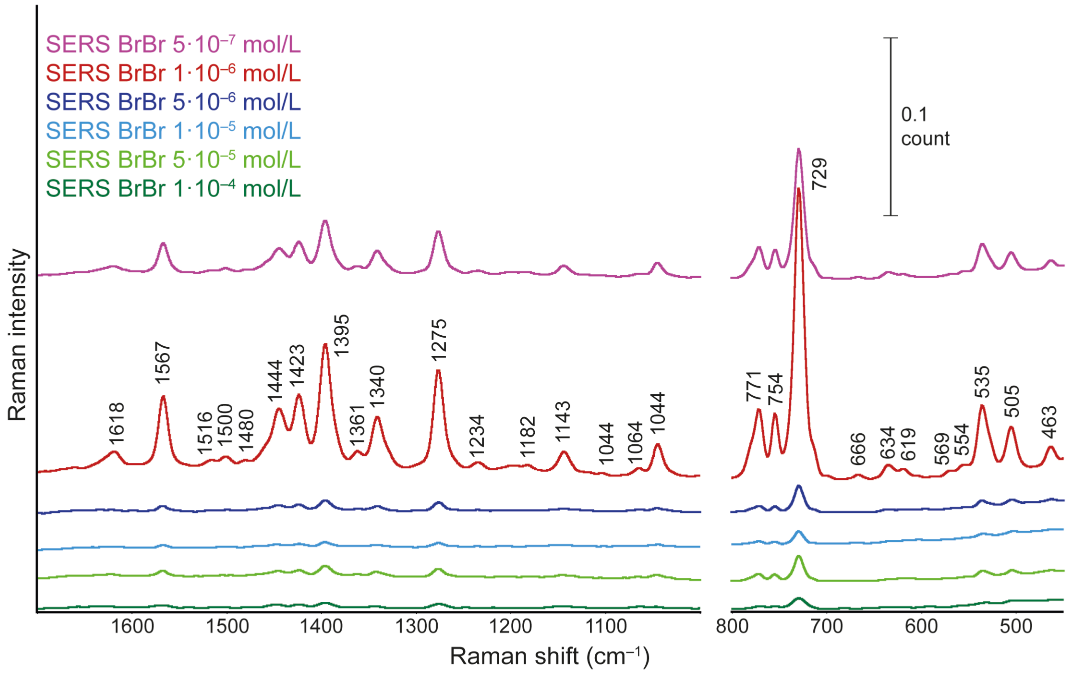

Figure S14. Berberine Ag-SERS spectra measured with different BrBr concentrations. The shown spectra are averages of ten independent spectra for each concentration. Spectra were measured using excitation wavelength of 1064 nm and are displayed on an off-set scale.

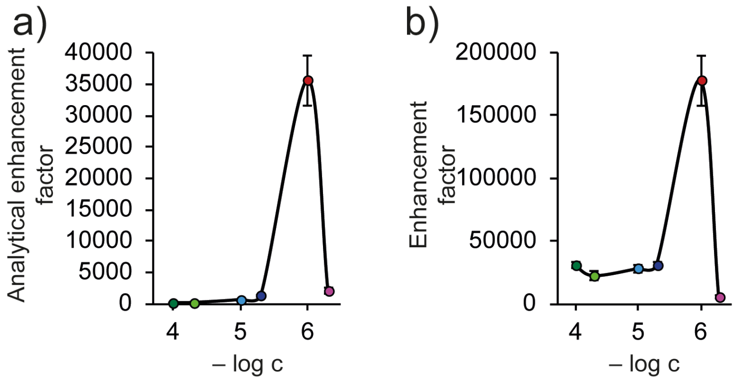

Figure S15. Concentration dependence of the enhancement factors values obtained from the Eq. 1 when using **a)** entire added concentrations of the BrBr, **b)** theoretical concentration value required for the complete surface coverage. The values were obtained from the spectra measured with an excitation wavelength of 532 nm. Error bars represent the standard deviation from all computations in both directions.

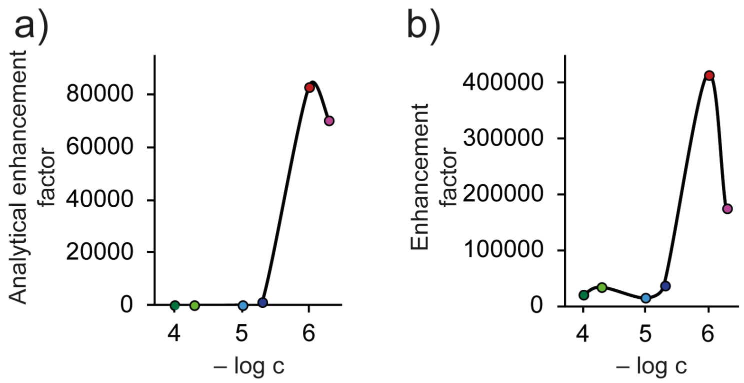

Figure S16. Concentration dependence of the enhancement factors values obtained from the Eq. 1 when using **a)** entire added concentrations of the BrBr, **b)** theoretical concentration value required for the complete surface coverage. The values were obtained from the spectra measured with an excitation wavelength of 1064 nm.

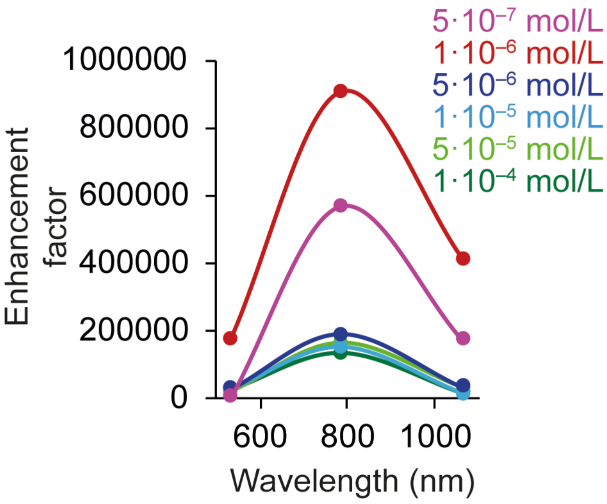

Figure S17. Excitation wavelength dependence of the enhancement factors computed using Eq. 1 for systems modified by different concentrations of BrBr.
